# Incidence of thrombotic microangiopathies in Quebec: insight from a laboratory centralizing ADAMTS-13 testing

**DOI:** 10.1186/s13023-022-02409-3

**Published:** 2022-08-04

**Authors:** Clémence Merlen, Emmanuelle Pépin, Ousmane Barry, Anik Cormier, Caroline Dubois, Anne-Laure Lapeyraque, Stéphan Troyanov, Georges-Etienne Rivard, Arnaud Bonnefoy

**Affiliations:** 1 Division of Hematology-Oncology, CHU Sainte-Justine, Université de Montréal, 3175, chemin de la Côte-Ste-Catherine, Montreal, QC H3T 1C5 Canada; 2 Department of Clinical Laboratory Medicine, OPTILAB Montréal-CHU Sainte-Justine, Montreal, QC Canada; 3 Division of Nephrology, CHU Sainte-Justine, Université de Montréal, Montreal, QC Canada; 4 Division of Nephrology, Hôpital du Sacré-Cœur, Université de Montréal, Montreal, QC Canada

**Keywords:** Thrombotic microangiopathies, Thrombotic thrombocytopenic purpura, ADAMTS-13, Incidence, Epidemiology, Prevalence

## Abstract

**Background:**

Thrombotic microangiopathies (TMA) are serious medical conditions requiring a prompt diagnosis to adapt treatment. The determination of ADAMTS-13 activity enables discriminating thrombotic thrombocytopenic purpura (TTP) from other forms of TMA. The purpose of this study was to provide an estimate of the incidence of TTP and TMA in the Canadian Quebec province using data collected from a laboratory centralizing ADAMTS-13 testing for the whole province.

**Results:**

From 2012 to 2019, 846 patients were evaluated for plasma ADAMTS-13 activity due to a suspicion of TMA. TTP was identified in 147 patients. Of these, 118 patients with a median age of 51.5 years and a male–female ratio of 1:1.4 had their first episode of TTP during the study period. The number of ADAMTS-13 tests performed and the number of patients with suspected TMA increased annually by 19% and 21% respectively. While the incidence of non-TTP TMA increased annually, that for TTP remained unchanged. This averaged 10.2 (95% CI 5.9–14.4) per million persons per year for suspected non-TTP TMA and 1.8 (95% CI 1.3–2.4) for confirmed TTP. The incidence rate of TMA other than TTP was higher in the age group 70–79 years (21.8; 95% CI 5.4–38.1) for females and in the age group 80–89 years (24.4; 95% CI 7.2–41.7) for males compared to other age groups. The incidence rate of TTP was higher in the age group 40–49 years (4.0; 95% CI 2.0–5.9) for women and in the age group 60–69 years (3.4; 95% CI 1.1–5.6) for men compared to other age groups.

**Conclusion:**

The analysis of centralized data measuring ADAMTS-13 activity allowed us to adequately establish the incidence rate and demographic characteristics of TMA, particularly TTP, in Quebec. TTP incidence remained stable while suspected non-TTP TMA steadily increased from 2012 to 2019.

**Supplementary Information:**

The online version contains supplementary material available at 10.1186/s13023-022-02409-3.

## Background

Thrombotic microangiopathies (TMA) are rare but life-threatening hematologic conditions mainly characterized by a severe thrombocytopenia, a microangiopathic hemolytic anemia (MAHA) and ischemic organ damage [1–3]. The predominant primary forms of TMA include thrombotic thrombocytopenic purpura (TTP), typical hemolytic uremic syndrome (HUS) and complement-mediated TMA (CM-TMA) also known as atypical HUS [2]. Although clinical manifestations overlap between TTP and other TMAs, recent advances in the understanding of the pathophysiology led to a better discrimination of TMA forms, which is of importance to guide initial therapy [4]. TTP is associated with a severe deficiency of ADAMTS-13 (a disintegrin and metalloproteinase with a thrombospondin type 1 motif, member 13) [5]. This deficiency leads to an accumulation of ultralarge von Willebrand factor (VWF) multimers and the unrestrained growth of VWF and platelet-rich thrombi occluding microvessels throughout the body [1]. TTP are mostly acquired forms due to the presence of ADAMTS-13 autoantibodies, whereas less than 5% of TTP are due to mutations in the ADAMTS13 gene (Upshaw-Schulman syndrome) [5, 6]. CM-TMA are generally associated with mutations affecting the function of proteins down-regulating the complement alternative pathway, although autoantibodies against complement-regulating factors such as anti-Factor H antibodies account for a significant proportion of CM-TMA. Typical HUS are most often triggered by Shiga Toxin-Producing *Escherichia coli* (STEC) [7]. In addition to these primary forms of TMA, secondary TMAs associated with different underlying causes of endothelial cell damages can occur. Aggressive treatment of malignancies and allogenic organ transplantation also increase the risk of secondary TMAs [8]. Because of overlapping clinical signs, the differential diagnosis of TTP and other TMAs remains challenging. Clinical point scores have been developed to help predict severe ADAMTS-13 deficiency in the absence of ADAMTS-13 activity testing. However, the low predictive value of these scores applied to unselected TMA reinforces the importance of early ADAMTS13 testing to discriminate TTP from other TMAs [9, 10].

Severe ADAMTS-13 deficiency, responsible for TTP, is defined by a plasma ADAMTS-13 activity ≤ 10% of the average activity found in the plasma of a healthy population [11, 12]. Therapeutic plasma exchange is rapidly initiated in the presence of TMA symptoms, most often before obtaining an ADAMTS-13 activity result. If TTP is confirmed by ADAMTS-13 activity ≤ 10%, presence of ADAMTS-13 autoantibodies and no family history suggestive of an Upshaw-Schulman syndrome, corticosteroid therapy with the addition of rituximab is recommended during both first and relapse episodes [13]. If ADAMTS-13 activity is > 10% and STEC-HUS excluded, the diagnosis is either CM-TMA or a secondary TMA. The latter may be drug-induced or triggered by cancer, malignant hypertension, transplantation or pregnancy. The therapeutic strategies in these cases aim to suppress the triggering element, treat the primary cause and provide supportive care. The diagnosis of CM-TMA is often difficult and time consuming as it involves functional, antigenic and genetic investigations to identify the factor causing the over-activation of the alternative complement pathway. Plasma exchange has shown no or little benefit to treat CM-TMA whereas therapeutic complement blockade at the level of C5 (e.g. eculizumab) has proven its therapeutic efficacy [14].

The information available on the incidence and prevalence of TMAs has so far been extrapolated from data obtained through national registries, reviews of the literature or multicenter retrospective analyses. These studies have reported an annual estimated incidence for TTP ranging from 1 to 6 per million population and an annual estimated prevalence of approximately 10–13 cases per million population [15–24]. In the province of Quebec, the supply of medical biology tests is organized according to geographical criteria and expertise. As of 2013 the Quebec Ministry of Health has mandated that all requests for ADAMTS-13 activity and anti-ADAMTS-13 antibody testing be performed at hemostasis laboratory of CHU Sainte-Justine (CHUSJ). This structure gave us the opportunity to comprehensively study the incidence and prevalence of TTP and other suspected TMAs in the second largest province of Canada with approximately 8.5 million persons in 2019 (22.6% of the Canadian population) [25].

## Material and methods

### Study design

The study was performed at the CHUSJ from April 30, 2012 through December 31, 2019. All consecutive requests for ADAMTS-13-activity testing received by the CHUSJ hemostasis laboratory during this period were considered. Only requests for testing plasma originating from a Quebec physician were included in the study. Exclusion criteria were as follows: requests from outside the province, requests for research or non-medical purpose and requests for a familial investigation. The study was approved by the Research Ethics Committee of the CHUSJ (#2019-2184).

### Data collection

Data on demographic characteristics (age, sex) and laboratory results (ADAMTS-13 activity and anti-ADAMTS-13 antibody titration) validated by the CHUSJ hemostasis laboratory since April 2012 were extracted from the laboratory information system (SoftLab version 4.0.8.3.7; SCC Soft Computer, Florida). Data on prior episodes of TMA as well as the time of sample collection relative to treatments were collected from a medical questionnaire completed by the requesting physician that came with each ADAMTS-13 request (see Additional file 1). ADAMTS-13 activity testing was available since April 2012 at the CHUSJ hemostasis laboratory. In 2013, the laboratory was designated as the exclusive supplier of the test in the province by the *Institut National d’Excellence en Santé et Services Sociaux* (INESSS), with unrestricted use by any Quebec referring hospital.

### Laboratory tests

#### Blood sampling

Peripheral venous blood samples were collected into 3.2% (0,109 M) buffered sodium citrate tubes. Platelet-poor-plasma (PPP) was obtained by double centrifugation at 2500 g for 10 min and was stored at − 80 °C until testing. Specimens collected outside the CHUSJ were sent on dry ice and promptly stored at − 80 °C until used for testing.

#### ADAMTS-13 activity measurement

ADAMTS-13 activity testing was routinely performed at least once a week or within 24 h of reception when urgent testing was requested. Activity was assessed with an in-house assay measuring the cleavage of an ADAMTS-13-specific fluorescent FRETS-VWF73 substrate (Peptide Institute, Osaka, Japan) [26] on a Synergy 4 microplate reader (BioTek, Winooski, VT).

### Definitions

We used the following terms to define the groups of patients studied.

TTP patients: patients with a clinically suspected TMA having a result of ADAMTS-13 activity ≤ 10% during the acute phase and before any plasma-based treatment and/or confirmed by the presence of anti-ADAMTS-13 antibodies or by medical notes from the treating physician who had ordered ADAMTS-13 testing, for patients previously diagnosed with TTP.

Non-TTP TMA patients: all patients with a clinical suspicion of TMA with a functional ADAMTS-13 activity at presentation > 10% prior to plasma treatment.

### Incidence rates and period prevalence

The annual incidence rates were calculated as the sum of patients with a first-time recorded suspicion of TMA including all TMA types, non-TTP TMA (as defined above) or confirmed TTP and divided by the Quebec mid-year estimated population of the related years. The Quebec population estimates were obtained from Statistics Canada (year 2012: 8.061 million; year 2013: 8.111; year 2014: 8.150; year 2015: 8.175; year 2016:8.225; year 2017: 8.302; year 2018: 8.401; year 2019: 8.501) [27]. Individuals with a documented history of TMA according to the medical questionnaire were not included in the incidence calculation. Each new case was also classified by age group in decades, gender and year of event in order to calculate the age-sex incidence rates. The prevalence of TTP measured the number of new and pre-existing TTP cases for every year divided by the population size at the time period mid-point.

### Statistical analyses

Statistical analyses and graphs were performed using IBM SPSS statistics version 27.0 and R version. 4.1.1 with the use of the tidyverse 1.3.0 package [28]. Demographic characteristics were calculated as median and interquartile range (IQR). Comparisons between patients with TTP vs patients with non-TTP TMA were analyzed using the Mann–Whitney U test (continuous variables) or χ^2^ test (categorical variables). A p-value of 0.05 or less was considered statistically significant and all tests were two-sided.

## Results

### Population and categorization of patients with suspected TMA

**Fig. 1:**
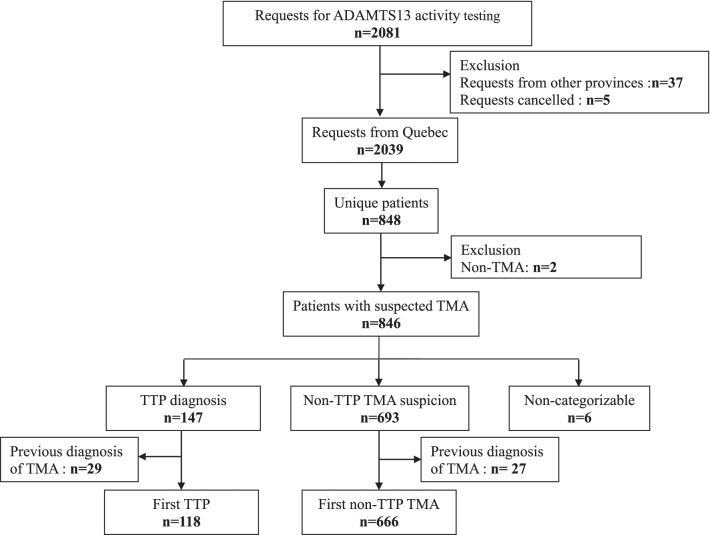
Flow chart of the study and patient categorization. *ADAMTS-13* a disintegrin and metalloprotease with thrombospondin type 1 repeats, member 13, *TMA* thrombotic microangiopathy, *TTP* thrombotic thrombocytopenic purpura

Over the study period, CHUSJ received 2081 requests for ADAMTS-13 activity measurement (Fig. 1). These requests included new investigations, as well as longitudinal monitoring of known TTP patients. For the province of Quebec, we performed 2039 analyses in 848 different patients. Two children were investigated for ADAMTS-13 activity due to a family history of TTP and were excluded from the incidence as their ADAMTS-13 activity was > 10%. The remaining 846 patients with suspected TMA constituted the total cohort. Among them, 6 (< 1%) with suspected TMA but an ADAMTS-13 activity > 10% could neither be categorized as TTP nor non-TTP TMA due to uncertainties regarding the timing of specimen collection in relation to plasma treatment. There were, 147/846 patients (17%) with confirmed TTP and 693/846 (82%) considered to have non-TTP TMA (based on clinical suspicion of TMA other than TTP). Among the TTP patients, 145 had severe deficiency of ADAMTS-13 activity at presentation, before any treatment and/or had anti-ADAMTS-13 antibody, confirming TTP. Two patients were placed in this category because of the explicit mention of diagnosis of TTP provided in the medical questionnaire. Previous TMA episodes were reported in 29/147 (20%) of patients with TTP and in 27/693 (4%) of patients with non-TTP TMA inside or outside the study period. Therefore, 118 patients with TTP and 666 with non-TTP TMA had their first episode of TMA during the study period.

### Trends in annual number of ADAMTS13 activity requests and patients

**Fig. 2:**
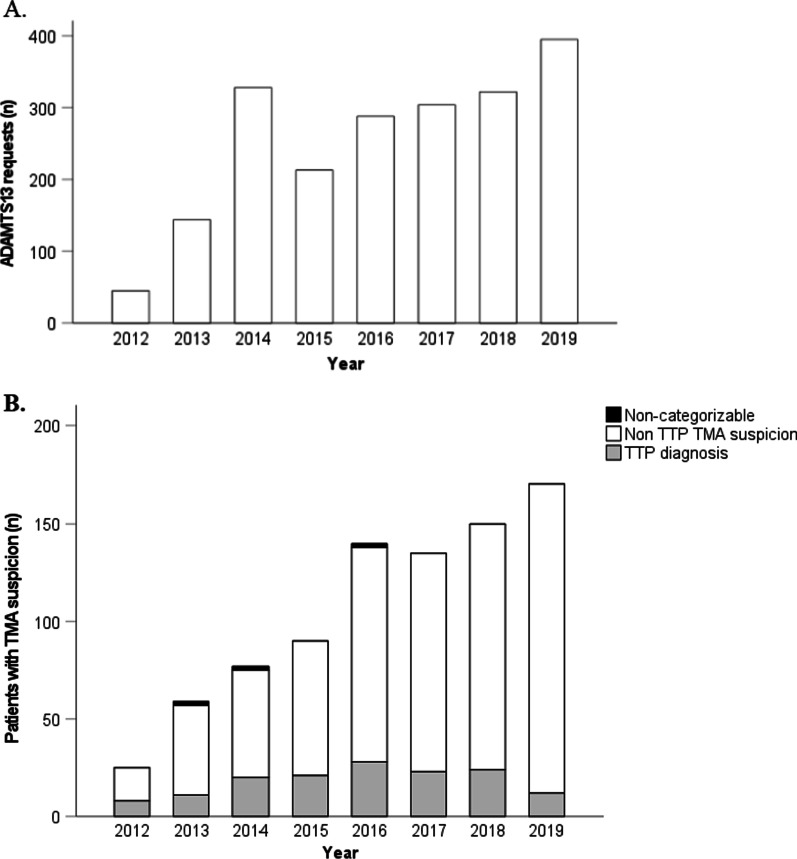
Trends in ADAMTS-13 testing and number of patients with clinically suspected thrombotic microangiopathy according to year. **a** Number of ADAMTS13 activity requests by year. **b** Number of patients with thrombotic microangiopathy other than TTP (non-TTP TMA) suspicion (white) and with TTP (grey) by year

Figure 2 represents the number of ADAMTS-13 activity tests performed and the number of patients with suspected TMA at the time of their first presentation according to year. Requests for ADAMTS-13 activity testing increased threefold from 2013 (the first complete year of the centralization of the test) to 2019 (Fig. 2a). The peak of requests observed in 2014 is attributed to 150 requests received as part of a follow-up schedule for a single refractory TTP patient (patient #252) hospitalized during that year at CHUSJ. When excluding this patient, the number of requests increased annually by an average of 18.7%. The number of new patients/year with suspected TMA (with or without prior episodes of TMA) also increased threefold from 2013 to 2019 (Fig. 2b). This represented a mean annual increase rate of 20.6%. This increase was mainly attributable to new non-TTP TMA patients/year as they represented 68% (n = 17/25) of TMA suspicion in 2013 and 93% (n = 158/170) in 2019. By contrast, the number of new TTP patients/year depicted a bell-shape curve, rising from 8/25 (32%) in 2012 to 28/140 (20%) in 2016 thereafter decreasing down to 12/170 (7%) in 2019.

### Demographic characteristics of patients at presentation

The proportion of females in the TTP (62%) and non-TTP groups (59%) did not differ significantly (p = 0.21). The patient’s age ranged from < 1 to 93 years (median age = 56 years; IQR = 32–69) in the non-TTP TMA group (n = 693) and from < 1 to 84 years (median age = 49 years; IQR = 35–63) in the TTP group (n = 147). In keeping with the age distribution of the Quebec population, 86% of non-TTP patients and 94% of TTP patients were older than 19 years.

**Fig. 3:**
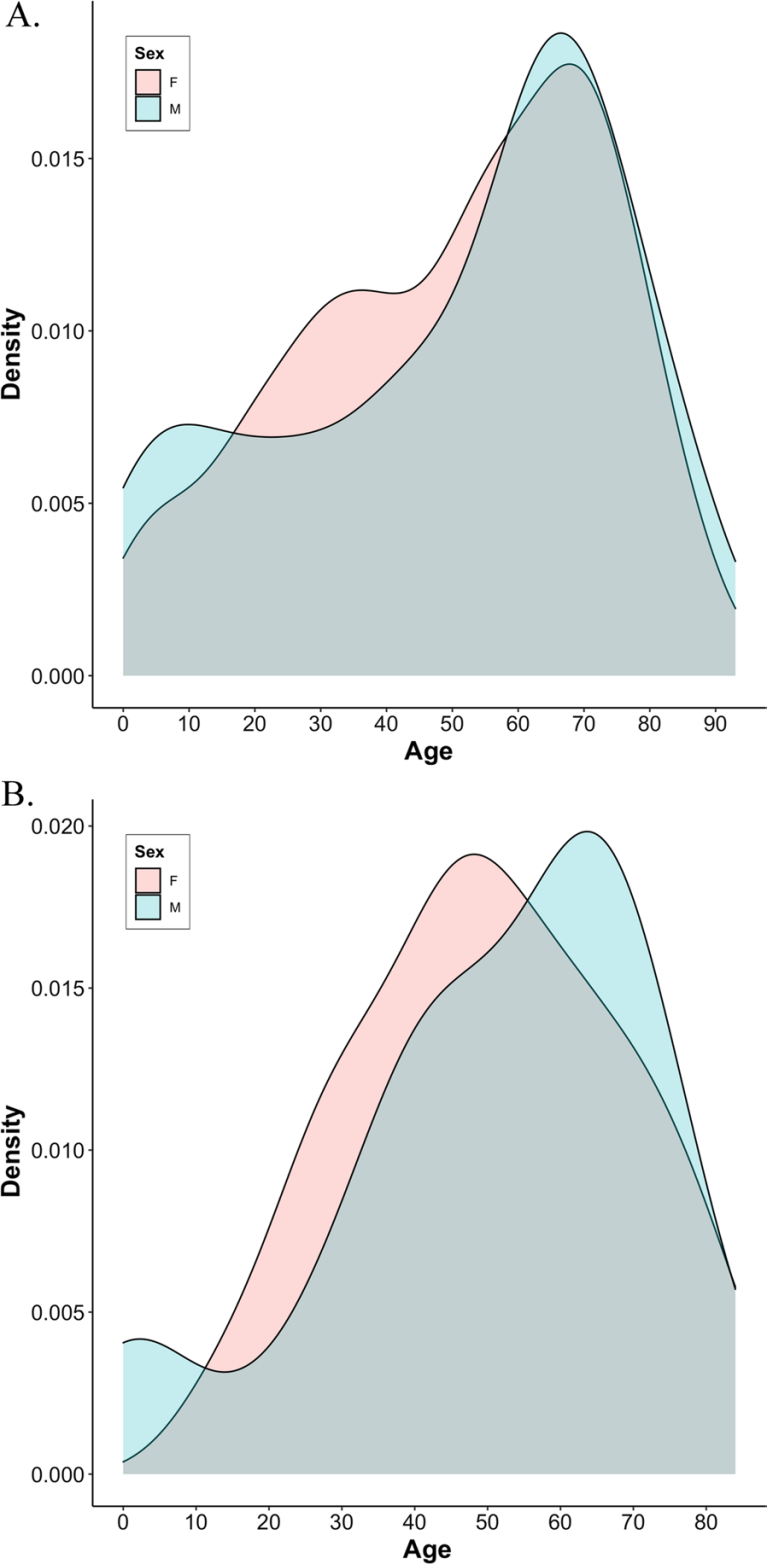
Age distribution of patients tested for ADAMTS-13 activity from 2012 to 2019 according to gender. **a** Patients with thrombotic microangiopathy other than TTP (non-TTP TMA). **b** Patients with TTP

Figure 3 represents the Gaussian kernel density estimation of the age distribution according to gender in newly diagnosed non-TTP (n = 666) (Fig. 3a) and TTP (n = 118) (Fig. 3b) TMA patients. The age distribution profiles for non-TTP and TTP males show a biphasic trend with a small peak in childhood and a main peak around 65 years of age. Adult females were predominantly counted in both non-TTP and TTP with a tendency to be younger than adult males. There was no significant difference between females and males median ages in both non-TTP (54.2 vs 58.0 years, respectively, p = 0.56) and TTP TMA patients (49.0 vs 56.5 years, respectively, p = 0.49).

### Incidence rates of thrombotic microangiopathies and thrombotic thrombocytopenic purpura

Annual incidence (number of cases per million of the population per year) was calculated for total suspected TMA, non-TTP TMA and TTP patients who had their first episode of TMA during the study period in relation to the general population (Table 1). For the period studied (2012–2019), the mean annual incidence was 12.0 (95% CI 7.4–16.6; median age = 56 years) for total TMAs, 10.2 (95% CI 5.9–14.4; median age = 56 years) for non-TTP TMA and 1.8 (95% CI 1.3–2.4, median age = 51.5 years) for TTP. The annual incidence of non-TTP TMA increased threefold from 2013 to 2019. In contrast, the annual incidence of TTP increased from 2013 to 2016 then decreased from 2017 to 2019.

**Table 1:** Annual incidence of thrombotic microangiopathies according to gender and year of diagnosis in Quebec

	Year 2012*	Year 2013	Year 2014	Year 2015	Year 2016	Year 2017	Year 2018	Year 2019
*Suspected TMA*								
Total (n = 786)	4.10	6.78	8.10	10.15	15.32	15.54	16.42	19.64
Female (n = 459)	5.18	7.60	9.28	12.17	18.16	16.56	20.66	22.32
Male (n = 327)	3.00	5.95	6.91	8.11	12.45	14.50	12.17	16.96
*Suspected non-TTP TMA*								
Total (n = 666)	3.16	5.67	6.26	8.19	12.52	13.13	13.92	18.35
Female (n = 390)	3.33	7.11	7.57	9.74	14.77	13.44	17.80	20.91
Male (n = 276)	3.00	4.22	4.93	6.64	10.25	12.81	10.02	15.78
*Supported TTP*								
Total (n = 118)	0.93	1.11	1.72	1.96	2.67	2.41	2.50	1.29
Female (n = 69)	1.85	0.49	1.71	2.43	3.39	3.12	2.85	1.41
Male (n = 49)	0	1.74	1.73	1.47	1.95	1.69	2.15	1.18

Annual incidence: number of new cases per million population per year; TMA: thrombotic microangiopathy; TTP: thrombotic thrombocytopenic purpura

*The incidence rate has been extrapolated for year 2012

**Fig. 4:**
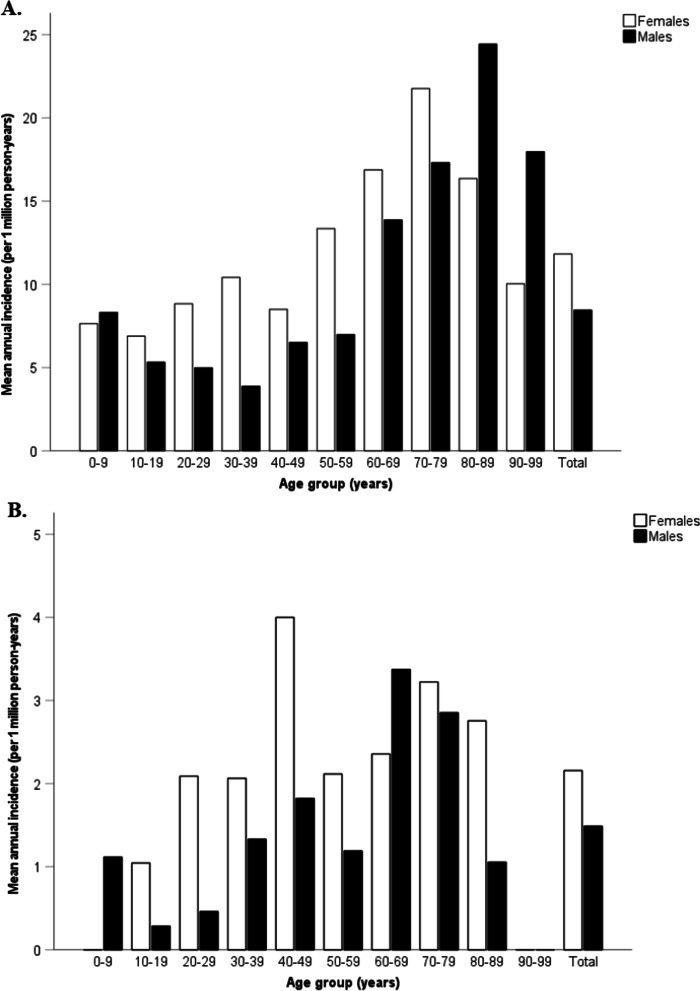
Mean annual incidence rate by age group and gender. **a** Clinically suspected thrombotic microangiopathy other than TTP (non-TTP TMA) (n = 666). **b** Confirmed TTP (n = 118). The incidence rate has been extrapolated for year 2012

Figure 4 depicts the age-specific annual incidence of non-TTP TMA and TTP according to gender. Over the study period, the incidence of non-TTP TMA were significantly higher for females (11.8, 95% CI 6.9–16.8) than for males (8.5, 95% CI 4.7–12.2; p = 0.001). In females, the incidence increased from 7.3 (95% CI 4.1–11.5) during childhood (0–19 years) to 21.8 (95% CI 5.4–38.1) at range 70–79 years, then decreased with increasing age. For males of the same group, two peaks were observed at ages 0–9 years (8.3: 95% CI 4.4–12.2) and at 80–89 years (24.4; 95% CI 7.2–41.7) (Fig. 4a). The incidence of TTP was also significantly higher in females (2.2, 95% CI 1.3–3.0) compared to males (1.5, 95% CI 0.9–2.1) (p = 0.001). A peak incidence was observed in the age group 40–49 years (4.0 cases per million females per year; 95% CI 2.0–5.9) for females and in the age group 60–69 years for males (3.4 per million males per year; 95% CI 1.1–5.6) with confirmed TTP (Fig. 4b).

### Prevalence of thrombotic thrombocytopenic purpura

ADAMTS-13 laboratory data can be used to determine the annual prevalence of TTP since this assay is required at the time of diagnosis, during patient follow-up and during recurrences. Conversely, these data are not relevant for calculating the prevalence of non-TTP TMA subjects since their ADAMTS-13 level is usually measured only once at the time of their first episode of TMA to exclude TTP (which corresponds to their incidence). The prevalence was estimated by dividing the annual number of TTP cases by the general population size or the total number of males or females (Table 2). Over the study period, the mean annual TTP prevalence (per million population) was 4.3 cases (95% CI 2.7–5.9) for the total population, 5.6 (95% CI 3.4–7.8) for female population and 3.0 (95% CI 1.7–4.2) for male population. Prevalence of TTP steadily increased among females from 2012 to 2019 in contrast with males for whom TTP prevalence plateaued from 2014.

**Table 2:** Estimated prevalence of thrombotic thrombocytopenic purpura according to gender and year in Quebec

	Year 2012*	Year 2013	Year 2014	Year 2015	Year 2016	Year 2017	Year 2018	Year 2019
*Supported TTP*								
Total	1.49	1.73	3.43	4.52	5.35	5.42	6.07	6.35
Female	2.96	1.72	3.66	5.36	7.26	7.68	7.36	8.93
Male	0	1.74	3.21	3.69	3.42	3.15	4.77	3.77

Prevalence: number of cases per million population per year. TTP: thrombotic thrombocytopenic purpura

*The prevalence rate has been extrapolated for year 2012

## Discussion

TMAs are associated with high morbidity. Even when properly managed, the mortality rate of TTP is up to 20%. Relapses and long-term effects of TMAs include depression, chronic kidney disease and cardiovascular diseases and require a continuing follow-up with long-term and expensive treatments [29].

We believe that this study constitutes an accurate description of TMA occurrence in Quebec. This was made possible by centralizing ADAMTS-13 activity testing.

The annual incidence of non-TTP TMA steadily increased between 2012 and 2019 with no difference between males and females. One must keep in mind that in the present study, the non-TTP TMA incidence is an approximation based on the postulate that ADAMTS-13 activity testing is a first line analysis prescribed for any patient with a suspected TMA, in order to differentiate TTP from any other non-TTP TMA. Several reasons may explain the increase of both ADAMTS-13 testing and non-TTP TMAs during the study period: (1) a growing awareness among physicians of the existence of TMAs and ADAMTS-13 testing [30]; (2) a better and unrestricted access to the test due to its inclusion in the province's official directory of medical tests [31]; (3) the requirement for the treating physician to exclude a diagnosis of TTP—i.e. to confirm a level of ADAMTS-13 activity level above 10%—in order to have access to the anti-C5 drug eculizumab for their patient with a suspected CM-TMA [32]; and (4) a true increase in non-TTP TMA. In recent years, new treatments for malignancies have become available, some of which are associated with an increased risk of TMA including vascular endothelial growth factor (VEGF) inhibitors and gemcitabine. In addition, the number of allogeneic stem-cell and solid organ transplantations have greatly increased [33] where TMA can be observed in graft-vs-host disease and with the use of calcineurin inhibitors.

The average incidence of TTP during the study period was 1.82 per million per year. This estimation is consistent with previous studies. In other Canadian provinces, the incidence rate was found to be 1 (Bristish Columbia) and 3.2 (Saskatchewan). Other countries reported annual incidences of 1.5 (France, Germany), 3.1 (Oklahoma, U.S.) and 6.0 (U.K.) [15, 17, 19, 20, 22]. This variability may result from demographic factors and definitions used to diagnose TTP [34]. In our study, the incidence of TTP evolved differently from that of non-TTP TMA. We observed a 2 to threefold increase in the incidence of TTP from 2013 to 2016 which plateaued from 2016 to 2018 then decreased in 2019. Noticeably, the temporal increase of TTP incidence is mainly seen in the female group. At this stage, it remains difficult to determine whether this variation is random or a significant epidemiological phenomenon. Potential interpretation bias should not be excluded: the initial increase and plateau could be due to the analysis by the laboratory of patients samples previously identified as TTP but for whom no ADAMTS-13 assay had been performed until 2016. The average annual prevalence of TTP (year 2012–2019) was 4.3 cases per million reflecting both acute and monitoring requests. This prevalence is lower than a previously reported estimation of 10–13 cases per million for TTP [17, 35], which could be due to differences in operational definitions between studies [36].

TMA can affect people of all ages and gender, but an average age at first presentation of 40 years and a female predominance (1 male for 2 to 3 female) has been previously reported for TTP in studies conducted in South Africa (15) and North American (Oklahoma) [19, 37]. In our study, the median age of non-TTP patients and TTP patients, who had no previous history of TMA was 56 years and 51 years respectively. We found a male–female ratio of 1:1.4 comparable to the ratio of 1:1.6 reported by a Canadian apheresis referral center (British Columbia) in patients with TTP [22]. In their study, Martin et al., found a median age at first presentation of 51 years, which corroborates our findings and suggest that differences in median age of patients with TTP between studies is due to demographic differences across countries. The predominance of females remained overall true when stratifying by age group, especially between the ages of 20 to 49 years. Indeed, the incidence was more than twofold higher in the age group of 30–39 years for females with non-TTP TMA and more than fourfold higher in the age group of 20–29 years for females with TTP compared to males. The clear trend for a higher incidence in females in the group of childbearing age was expected because pregnancy is a known risk factor of TMA [38]. However, non-TTP TMA incidence in females fades with increasing age whereas it increases in males beyond the age of 80 years. TMA affects a significant proportion of elderly patients with a peak incidence over 60 years of age in this study. Elderly patients present more comorbidities (mental alteration, stroke, renal dysfunction, cancer) than the rest of the population, which contributes to delay the diagnosis of TMA. They also seem to develop more TTP and secondary TMA associated with cancer or medication [39]. A better awareness of TMA-related complications could contribute to a faster diagnosis and a better management of these elderly patients whose greater frailty contributes to an increased mortality rate.

In contrast to the TTP TMA group for which severe deficiency of ADAMTS-13 activity is confirmatory, our estimates of the non-TTP TMA incidence may be overestimated. Indeed, non-TTP TMAs included true primary or secondary TMA and any other disease with TMA-like clinical presentation such as severe hypertension, severe sepsis with coagulopathy or mechanical causes [40]. Accordingly, TTP cases represented a mean of 20% per year of suspected TMA cases, proportion which is very close but lower to that obtained in the French, the Oklahoma and the Japanese registries who reported respectively a proportion of 24%, 23% and 21% for TTP among all TMA cases [17, 19, 41, 42].

The main limitations of our study are the lack of data regarding the medical reasons for TMA suspicion and the potential for misclassification of TMA. Patient registries allow access to reliable epidemiological data and offer the advantage of collecting standardized information as well as pooling data over time [43, 44]. However, the regulated structure and complex maintenance of registries can hinder the long term as well as for the recruitment and conservation of enrolled patients. Furthermore, an indirect selection bias can occur in the use of data from registries when access to the program is not mandatory. Missing data due to a low or inconsistent participation may also limit registry studies [45, 46], especially in rare diseases. The centralization of ADAMTS-13 activity testing in one reference center has the advantage of taking into account every case investigated in a given territory. Although the CHUSJ was designated as the only specialized center performing the activity assay, it is possible that some requests were sent to centers outside the province, although this would be rare for the following reasons: (1) the financial constraint imposed by the Ministry of Health in the event of non-compliance with this service corridor; (2) the optimization of turnaround time and testing cost made possible by the centralization of the analysis. For this study, we purposely stopped data entry in December 2019, so as not to include any patients infected by the SARS-CoV-2 (severe acute respiratory syndrome coronavirus 2) responsible for Coronavirus disease-2019 (COVID-19) as several reports described TMA-like presentations in infected patients [46]. The impact of the pandemic on the incidence of TMAs in Quebec is currently being investigated.

In conclusion, this study is useful both for the health system and for the management of patients affected by these rare and serious diseases. Further investigations on clinical presentation and underlying conditions associated with TTP and non-TTP TMA is needed to provide a complete clinical picture of TMA in the province of Quebec. The establishment of a regional registry may help to refine these results.

## Supplementary Information

**Additional file 1**. Medical questionnaire for analysis of ADAMTS-13.

## Data Availability

The datasets generated for this study are stored on a secure server at CHU Ste-Justine and can be shared with the authorization of the CHU Ste-Justine authorities.

## Abbreviations

ADAMTS-13: A disintegrin and metalloproteinase with a thrombospondin type 1 motif, member 13
CM-TMA: Complement mediated thrombotic microangiopathy
CHUSJ: CHU Sainte-Justine
COVID-19: Coronavirus disease-2019
HUS: Hemolytic uremic syndrome
INESS: Institut National d’Excellence en Santé et Services Sociaux
IQR: Interquartile range
MAHA: Microangiopathic hemolytic anemia
PPP: Platelet-poor-plasma
SARS-COV-2: Severe acute respiratory syndrome coronavirus 2
STEC: Shiga Toxin-Producing *Escherichia coli*
TMA: Thrombotic microangiopathy
TTP: Thrombotic thrombocytopenic purpura
VEGF: Vascular endothelial growth factor
VWF: Von Willebrand factor
