# Supplementary material for: Incidence of thrombotic microangiopathies in Quebec: insight from a laboratory centralizing ADAMTS-13 testing
Source: Orphanet J Rare Dis. 2022 Aug 4;17:308. doi: 10.1186/s13023-022-02409-3 (PMC9351225; doi:10.1186/s13023-022-02409-3)
Supplement: Supplementary file 1 — Additional file 1. Medical questionnaire for analysis of ADAMTS-13. [file 13023_2022_2409_MOESM1_ESM.pdf]

CHU Sainte-Justine
Le centre hospitalier universitaire mère-enfant
Université de Montréal

F- 726# INV.: 30006216 (RÉV. 2014-02)

ANALYSIS OF ADAMTS-13 IN CASES OF TROMBOTIC MICROANGIOPATHIES

ATTENTION: Filing of shaded zones is required

ANALYSIS OF ADAMTS-13 IN CASES OF TROMBOTIC MICROANGIOPATHIES

3175, Côte Sainte-Catherine Road, Montreal, QC H3T 1C5 (514) 345-4931 ext. 5681

Requesting Institution/Unit : ______________________

Address: Civic number ______ Street ______________

Province/Country ______________________

Postal code: ____________

Phone number: ______________ FAX: ______________

Requesting Physician : ______________________

Sampling Date: Y/M/D ____________ **Time** ________

Sampled By: ______________________ **STAT** ☐

Patient Information

Last Name, First Name

Gender : F ☐ M ☐

Healt care Institution (specify)
Medicare card # / Health facility file #
D.O.B. :

or

Stamp the patient's Health Care Institution card

CLINICAL INFORMATION FORM TO ATTACH TO SAMPLES

SUSPECTED PATHOLOGY :

» Thrombotic thrombocytopenic purpura (TTP) : ☐
» Hemolytic-uremic syndrome (HUS) : Typical ☐ Atypical ☐

PRIOR EPISODES of thrombotic microangiopathy: ☐NO ☐YES Specify dates: Y/M/D ____________

ASSOCIATED PATHOLOGIES :

Pregnancy ☐ Weeks preg.________ ☐ Cancer ☐ Infection ☐ Transplant
Medication ☐ Specify : __________ ☐ Other Specify :______________________

CLINICAL CONTEXT THAT CORRESPONDS TO AN ACUTE STAGE :

» Fever : ☐NO ☐YES
» Neurological signs : ☐NO ☐YES Specify : ______________
» Abdominal signs : ☐NO ☐YES Specify : ______________

BIOLOGICAL PARAMETERS :

» Hemolytic anemia : ☐NO ☐YES
» Thrombopenia : ☐NO ☐YES

TREATMENT :

Time of collection in relation to treatment :______________________________

If treatment, which one?:______________________________

____

SAMPLING PROCEDURES AND SHIPMENT OF BLOOD SAMPLES

- Collect peripheral venous blood samples using 4,5 ml citrate tubes containing 3,2% (0,109 mM) sodium citrate solution. Except for specific cases, collect blood before treatment, such as plasma transfusion and / or plasma exchange.

Note: Do not collect samples using EDTA or HEPARIN.

- Centrifuge the blood samples at 2 500g or more for 10 minutes at between 18 and 25°C
- Decant plasma into several aliquots (minimum of 3) of at least 500µL each
- Freeze the aliquots of plasma at -20°C or lower until they are ready to be shipped on dry ice
- **All of the aliquots must be clearly identified (Patient's family name, given name, birth date, sample date and time)**
- **The aliquots must be shipped along with the clinical information form (F-726)**
- **Ship the aliquots on dry ice to the laboratory:** CHU Sainte-Justine
Hemostasis Laboratory, 2nd floor, Unit 6, Room 2610, C/O Anik Cormier
3175, Côte Sainte-Catherine Road
Montreal, QC, Canada H3T 1C5

Information: (514) 345-4931 #7170

On the package
- Write the laboratory's complete address
- Write the name of the originating hospital and laboratory (you)
- Indicate on the package how many samples it contains
- Insert the requisition forms (F-726) in a sealed Ziploc bag and place them in a separate compartment from the samples
